# Assessment of Thyroid Function in Chronic Kidney Disease Patients at King Abdulaziz Medical City

**DOI:** 10.7759/cureus.73180

**Published:** 2024-11-06

**Authors:** Azizah B Hafed, Roaa K Abdulkareem, Atheer M Almalki, Jana I Alradadi, Amjad Aldosari, Zubaida H Khan

**Affiliations:** 1 Anatomy, King Saud bin Abdulaziz University for Health Sciences, Jeddah, SAU; 2 Medicine, King Abdulaziz University, Jeddah, SAU; 3 Medicine, Ibn Sina National College for Medical Studies, Jeddah, SAU; 4 Medicine, Taif University, Taif, SAU

**Keywords:** chronic kidney disease (ckd), ckd stages, hypothyroidism, renal function parameters, thyroid dysfunction, thyroid function tests

## Abstract

Background

Chronic kidney disease (CKD) is a progressive and irreversible condition in which the kidneys lose their ability to perform synthetic, excretory, and metabolic functions. CKD is associated with various pathophysiological conditions that impact multiple organs, including the thyroid gland, which primarily secretes triiodothyronine (T3) and thyroxine (T4). This study aims to assess thyroid function in patients with CKD and explore the relationship between renal disease severity and thyroid function.

Methods

This retrospective study included 200 patients with CKD who were admitted to King Abdulaziz Medical City Hospital, a tertiary center in Jeddah, Saudi Arabia, between 2016 and 2023. Patients were selected based on their age and health status. A predesigned questionnaire was used to collect key demographic, renal, and thyroid function test data, which were then analyzed using IBM SPSS Statistics for Windows, Version 20.0 (Released 2011; IBM Corp., Armonk, NY, USA).

Results

Out of the 200 CKD patients analyzed in this study, 120 (60%) were male and 80 (40%) were female. The results showed that 73.4% (n = 149) had normal thyroid function (euthyroidism). Thirty-nine patients (20.5%) had hypothyroidism, while only two patients (1.1%) had hyperthyroidism. Thyroid-stimulating hormone (TSH), FT3, and FT4 levels were measured across various stages of CKD. Hypothyroidism was most prevalent in stages 5 (30.8%) and 4 (23.1%) based on FT3 levels. It was highest in CKD stage 5 (38.5%) and stage 3 (30.8%) based on T4 levels. Hyperthyroidism was observed in CKD stages 3 (50.0%) and 5 (50.0%) based on TSH levels.

Conclusions

Patients with CKD exhibit fluctuations in T3 and TSH levels, with thyroid dysfunction increasing in parallel with the severity of renal disease.

## Introduction

The frequency and incidence of chronic kidney disease (CKD) are rising globally. In the United States, the National Health and Nutrition Examination Survey reports a CKD prevalence of 11.7% [1]. In the Kingdom of Saudi Arabia, CKD has become a major health concern, with approximately 20,000 dialysis patients and 9,810 kidney transplant recipients receiving follow-up care [1]. The age-standardized prevalence of CKD in Saudi Arabia is estimated at 9,892 per 100,000 people (stages 1-5), excluding renal replacement treatments. This rate exceeds those in Western Europe (5,446/100,000) and North America (7,919/100,000) [1].

CKD is a progressive, irreversible disease that impairs the kidney’s excretory, synthetic, and metabolic functions [2]. The classification and definition of CKD have evolved over time. According to the latest international guidelines, CKD is defined by a glomerular filtration rate (GFR) of less than 60 mL/min per 1.73 m² for at least three months and/or the presence of kidney damage markers, such as proteinuria or albuminuria, for the same duration [3]. CKD is associated with several pathophysiological conditions that affect multiple organs, including the thyroid gland, which primarily secretes triiodothyronine (T3) and thyroxine (T4) [2]. Thyroid hormones significantly influence metabolism, growth, and protein synthesis regulation [2]. Impaired thyroid function commonly manifests as fatigue, edema, shortness of breath, and dizziness, symptoms frequently observed in CKD, which may lead to unrecognized hypothyroidism in these patients [4]. As a result, such patients may require thyroid treatment [5].

CKD alters thyroid function in various ways, such as reducing tissue levels of thyroid hormones, increasing iodine reserves in the thyroid, modifying peripheral hormone metabolism, decreasing circulating thyroid hormone levels, and reducing binding to carrier proteins [6]. Interestingly, serum thyroid-stimulating hormone (TSH) does not increase in CKD patients despite low T3 and T4 levels [6]. Patients with CKD and hyperthyroidism, however, exhibit appropriate TSH suppression [6].

Additionally, impaired thyroid function has been linked to increased CKD risk. A decrease in iodothyronines is associated with reduced renal perfusion, GFR, and altered tubular reabsorption, which results in decreased water excretion [7]. Recent studies have also highlighted the role of inflammation in thyroid dysfunction in CKD patients. Inflammation contributes to various diseases, including thyroid disorders and stress-related illnesses such as sepsis, CKD, and malnutrition [7]. Moreover, patients with a lower GFR face higher cardiovascular morbidity and mortality, which may be exacerbated by hypothyroidism, leading to conditions such as atrial fibrillation, heart failure, and ischemic heart disease [8].

A cross-sectional study conducted over one month on stage 5 CKD patients revealed that 74.5% were euthyroid, 24.4% had hypothyroidism, and 3.3% had subclinical hyperthyroidism [9]. In Khartoum, thyroid nodules and cysts were the second most common findings in CKD patients undergoing hemodialysis, with an incidence of thyroid goiters at 21.57% [10].

In Saudi Arabia, a cross-sectional study by the Nephrology Division of the Security Forces Hospital between January 2015 and February 2018 included 255 individuals with CKD. The study found that 166 participants had no hypothyroidism, 43 had subclinical hypothyroidism, and 46 had hypothyroidism [11]. Hypothyroidism was observed in 34.9% of patients with CKD, including dialysis patients, while 17.66% of patients were excluded [11]. Another study conducted at the Al Jabr Kidney Center in 2022 involving 99 patients showed that 5.1% had high TSH, 9.1% had low TSH, and 85.9% were euthyroid [8,11].

Despite the high incidence of CKD in Saudi Arabia, little is known about the prevalence of thyroid disorders in this population. This study was conducted to better understand this issue, as it is crucial for clinicians to manage patients with impaired kidney or thyroid function. Therefore, we aimed to investigate the relationship between CKD and thyroid function.

## Materials and methods

Study design, setting, and participants

This retrospective study utilized data from patient records in the “Best Care” electronic system, identified by the patient’s medical record number. Relevant data, including follow-ups and laboratory blood tests, were collected from the King Abdulaziz Medical City Hospital, a tertiary center in Jeddah, Saudi Arabia, spanning from 2016 to 2023. Convenience sampling was employed, focusing on accessible CKD patients from this period. A total of 1,140 patients were diagnosed with CKD, of whom 200 met the inclusion criteria.

The inclusion criteria required patients to be ≥18 years old, diagnosed with any stage of CKD, and have impaired thyroid function. Exclusion criteria included patients with missing data, pregnant women, those on medications that alter thyroid function, individuals with systemic diseases such as cancer or liver disease, patients undergoing chemotherapy, and those with acute illnesses, recent surgeries, or burns. Patients primarily diagnosed with thyroid disease were also excluded. Among the eligible patients, 24 were on hemodialysis, and three were on peritoneal dialysis; these patients were excluded. The remaining patients, not on dialysis or with undetermined dialysis status, were included in the study.

Data collection

A data collection sheet was created using Excel (Microsoft Corporation, Redmond, WA, USA) to gather essential information. The first section included demographic details such as date of birth, gender, and BMI, which was classified as normal (18.5-24.9 kg/m²) according to WHO criteria. The second section comprised laboratory investigations, including complete blood count, fasting blood glucose, renal function tests, electrolytes, free thyroxine (FT3 and FT4), and TSH levels. The third section focused on medical history, including comorbidities (cardiovascular disease (CVD), hypertension, type 1 and 2 diabetes mellitus, and dyslipidemia) and family history of thyroid diseases.

Data on the stages of CKD were obtained from the nephrology department’s database, following Kidney Disease: Improving Global Outcomes (KDIGO) guidelines. Patients were classified into five stages (1-5), with an additional “unspecified” category for patients diagnosed with CKD but lacking a recorded stage.

Euthyroidism was defined by normal TSH, free T4, and free T3 levels: TSH between 0.6 and 5.5 mIU/L, FT4 between 9 and 19 pmol/L, and FT3 between 2.3 and 4.1 pg/mL. Hypothyroidism was characterized by elevated TSH levels (>5.5 mIU/L) alongside low FT3 and FT4 levels. Hyperthyroidism was defined as low TSH levels with elevated FT4 and/or FT3 levels.

Ethics approval

The study was approved by the Institutional Review Board of the Clinical Research Committee at King Abdullah International Medical Research Center in Jeddah, Saudi Arabia (IRB/1981/23). All procedures adhered to the ethical standards established by the institutional research committee.

Data analysis

All statistical analyses were performed using IBM SPSS Statistics for Windows, Version 20.0 (Released 2011; IBM Corp., Armonk, NY, USA), in accordance with the manufacturer’s instructions. Descriptive analyses for categorical data were presented as frequencies and bar charts. Continuous data for normally distributed variables were described using the mean and standard deviation. Chi-square, Fisher’s exact, and t-tests were employed to analyze categorical data. A p-value of <0.05 was considered statistically significant.

The key variables in this analysis include both demographic and clinical characteristics related to thyroid and kidney functions. Age was recorded in years, and BMI was calculated as weight in kilograms divided by height in meters squared (kg/m²). Serum creatinine concentration (mg/dL) was measured to assess kidney function and correlated with the GFR, calculated from the urine albumin-to-creatinine ratio. Blood urea nitrogen (BUN), another indicator of kidney function, was measured in mg/dL. Glucose metabolism markers, including fasting blood glucose (mmol/L) and HbA1c (%), were obtained as markers of long-term glycemic control. Thyroid function was evaluated using TSH levels (mIU/L) and thyroid function tests (TFT), which include T3 and T4 levels (nmol/L). Additionally, blood protein levels, such as albumin (g/L) and gamma globulin (%), as well as serum electrolytes (Na, K, and Ca) (mmol/L), were measured to monitor electrolyte balance and kidney function.

## Results

The study population consisted of 200 patients aged over 18 years, with a mean age of 63.4 ± 16.09. Among the patients, 60% (n = 120) were men, and 40% (n = 80) were women. The mean BMI was 29.2 ± 5.80, with 1.5% (n = 3) classified as underweight, 20.5% (n = 41) with normal weight, 27% (n = 54) overweight, and 43.5% (n = 8) obese.

**Figure 1:**
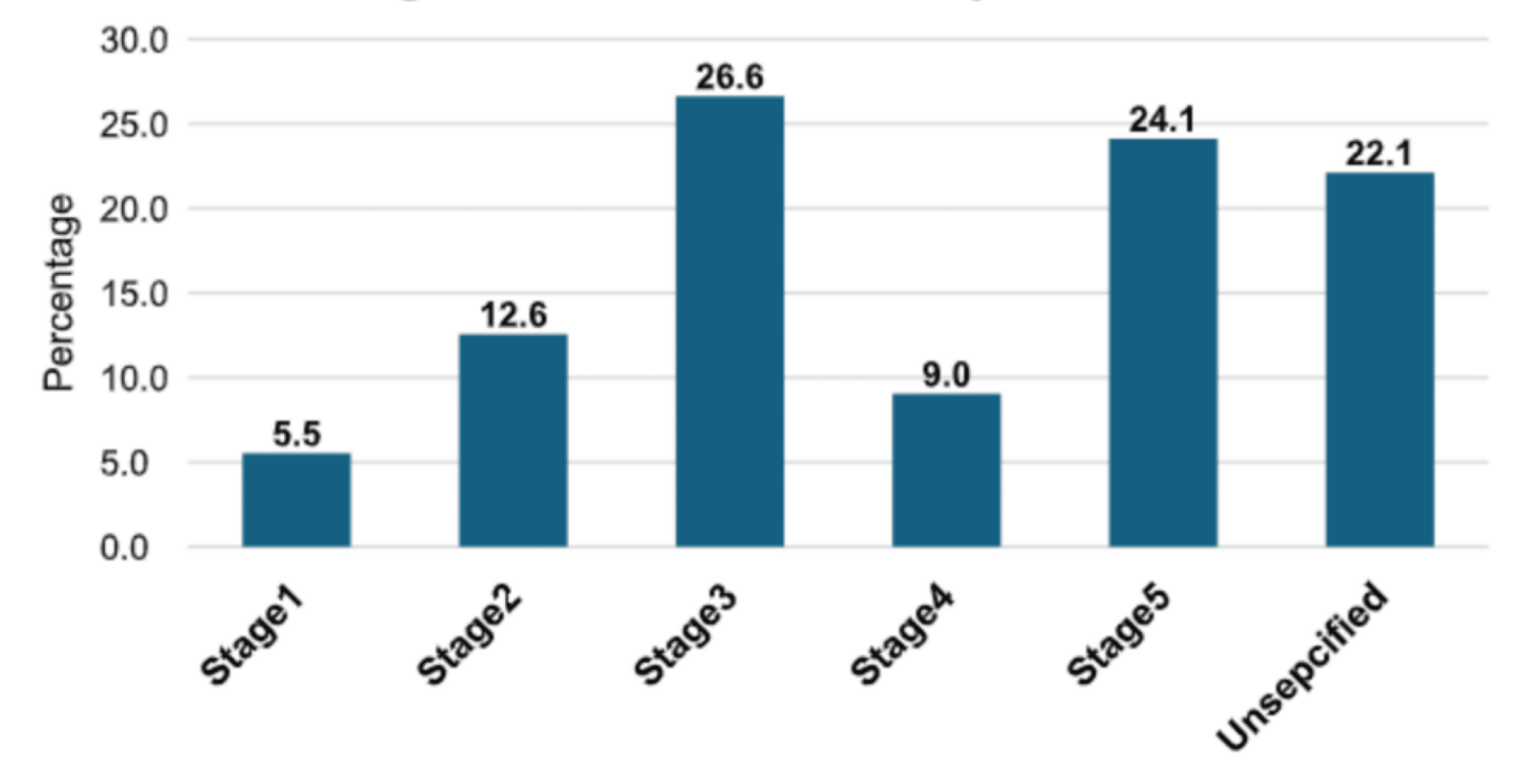
Stages of CKD among patients CKD, chronic kidney disease

Patients were classified into five stages of CKD based on nephrology department data: stage 5 (24.0%, n = 48), stage 4 (9.0%, n = 18), stage 3 (26.5%, n = 53), stage 2 (12.5%, n = 25), and stage 1 (5.5%, n = 11). The remaining 22.5% (n = 45) had unidentified stages (Figure 1). The majority of patients had stage 3 renal failure (26.5%, n = 53), followed by stage 5 (24.0%, n = 48).

Of these patients, 78.4% (n = 149) had normal thyroid function (euthyroidism). In contrast, 20.5% (n = 39) exhibited hypothyroidism, and a small proportion (1.1%, n = 2) had hyperthyroidism. These findings highlight the co-occurrence of thyroid dysfunction in CKD patients, underscoring the importance of considering thyroid health in CKD management. Monitoring thyroid function alongside CKD treatment is crucial for optimizing overall health in this patient population.

Thyroid function was analyzed by measuring free T4 and TSH levels at various CKD stages. Hypothyroidism was most common in stage 5 (30.8%, n = 4) and stage 4 (23.1%, n = 3) when considering T3 levels. However, hypothyroidism was most prevalent in stage 5 (38.5%, n = 5) and stage 3 (30.8%, n = 4) for T4 levels. Hyperthyroidism was observed in patients with stages 3 (50.0%, n = 1) and stage 5 (50.0%, n = 1) based on TSH levels. Euthyroidism showed varying prevalence across CKD stages, most notably in stage 3 (26.5%, n = 43) for T4 levels and in stages 3 (28.4%, n = 42) and 5 (23%, n = 34) for TSH levels. These findings emphasize the diverse thyroid dysfunction observed in CKD and the importance of considering thyroid function in CKD management (Table 1).

**Table 1:** Distribution of CKD patients according to stages Fisher’s exact test was used to analyze the distribution of thyroid dysfunction across different stages of CKD. CKD, chronic kidney disease; FT3, triiodothyronine; FT4, thyroxine; TSH, thyroid-stimulating hormone

Thyroid profiles	Stages of CKD						
	Stage 1	Stage 2	Stage 3	Stage 4	Stage 5	Unspecified	p-value
T3 level							
Hypothyroidism (n, %)	1 (7.7)	0 (0.0)	2 (15.4)	3 (23.1)	4 (30.8)	3 (23.1)	0.252
Euthyroidism (n, %)	1 (1.6)	11 (18.0)	12 (19.7)	6 (9.8)	14 (23.0)	17 (27.9)	
T4 level							
Hypothyroidism (n, %)	0 (0.0)	0 (0.0)	4 (30.8)	2 (15.4)	5 (38.5)	2 (15.4)	0.476
Euthyroidism (n, %)	9 (5.6)	21 (13.0)	43 (26.5)	14 (8.6)	36 (22.2)	39 (24.1)	
TSH level							
Hyperthyroidism (n, %)	0 (0.0)	0 (0.0)	1 (50.0)	0 (0.0)	1 (50.0)	0 (0.0)	0.585
Euthyroidism (n, %)	8 (5.4)	17 (11.5)	42 (28.4)	11 (7.4)	34 (23.0)	36 (24.3)	
Hypothyroidism (n, %)	2 (5.1)	7 (17.9)	7 (17.9)	6 (15.4)	10 (25.6)	7 (17.9)	

Several parameters were examined across thyroid status groups. The GFR was reduced in CKD patients, but no significant difference (p = 0.555) was observed across thyroid conditions. Patients with hyperthyroidism had an average GFR of 26.0 ± 12.73 mL/min/1.73, euthyroid patients had an average of 37.6 ± 25.41 mL/min/1.73, and hypothyroid patients had an average of 33.6 ± 22.18 mL/min/1.73. Serum creatinine levels (µmol/L) showed that patients with hyperthyroidism had an average of 315.0 ± 243.24 µmol/L, euthyroid patients had an average of 263.6 ± 249.04 µmol/L, and hypothyroid patients had an average of 273.8 ± 229.06 µmol/L. Regarding albumin levels (g/dL), patients with hyperthyroidism had an average of 38.0 ± 2.83 g/dL, euthyroid patients had a slightly higher average of 40.3 ± 4.09 g/dL, and hypothyroid patients had a lower average of 37.4 ± 6.47 g/dL. Statistical analysis revealed significant differences in albumin levels (p < 0.003). BUN levels were significantly elevated in hyperthyroid patients, with an average of 80.8 ± 63.99 mg/dL. In contrast, patients with hypothyroidism had significantly lower BUN levels, averaging 11.4 ± 6.31 mg/dL and 11.2 ± 7.16 mg/dL, respectively (p < 0.001). These results highlight the impact of thyroid health on kidney function-related parameters (Table 2).

**Table 2:** Association between TSH level and renal function test ANOVA test was used to analyze the association between TSH levels and renal function parameters. BUN, blood urea nitrogen; GFR, glomerular filtration rate; TSH, thyroid-stimulating hormone

Renal function tests	TSH	Mean	SD	95% CI		F	p-value
GFR (mL/min/1.73)	Hyperthyroidism	26	12.73	-88.36	140.36	0.591	0.555
	Euthyroidism	37.6	25.41	33.45	41.68		
	Hypothyroidism	33.6	22.18	26.37	40.75		
Serum creatinine level (µmol/L)	Hyperthyroidism	315	243.24	-1,870.5	2,500.5	0.067	0.935
	Euthyroidism	263.6	249.04	223.24	303.87		
	Hypothyroidism	273.8	229.06	199.54	348.05		
Albumin [A] (mg/dL)	Hyperthyroidism	38	2.83	12.59	63.41	5.906	0.003
	Euthyroidism	40.3	4.09	39.63	40.95		
	Hypothyroidism	37.4	6.47	35.34	39.53		
BUN (mg/dl)	Hyperthyroidism	80.8	63.99	-494.21	655.71	74.671	<0.001
	Euthyroidism	11.4	6.31	10.35	12.40		
	Hypothyroidism	11.2	7.16	8.91	13.55		

Among the CKD patients, 81% (n = 167) had normal thyroid function, while 18.5% (n = 33) were hypothyroid. In patients with CVD, the percentage with normal thyroid function dropped to 63.6% (n = 21), while the proportion with abnormal thyroid function increased to 30.3% (n = 10) with hypothyroidism and 6.1% (n = 2) with hyperthyroidism (p = 0.006). The presence of type 1 diabetes mellitus did not significantly affect TSH levels (p > 0.99), with all type 1 diabetes patients exhibiting normal thyroid function. No significant difference in TSH levels was found between individuals with and without type 2 diabetes mellitus (p = 0.677). Among patients without type 2 diabetes mellitus, 81.3% (n = 52) had normal thyroid function, while 18.8% (n = 12) were hypothyroid, and none had hyperthyroidism. In contrast, among patients with type 2 diabetes mellitus, 77% (n = 97) were hypothyroid, 21.4% (n = 27) were euthyroid, and only 1.6% (n = 2) were hyperthyroid. The presence or absence of hypertension, dyslipidemia, or type 2 diabetes mellitus did not significantly affect TSH levels (Table 3). 

**Table 3:** Associations between TSH level and CVD, T1DM, T2DM, hypertension, and dyslipidemia Fisher’s exact test was applied to analyze the associations between thyroid status and various comorbid conditions. CVD, cardiovascular disease; T1DM, type 1 diabetes mellitus; T2DM, type 2 diabetes mellitus; TSH, thyroid-stimulating hormone

Condition	Hyperthyroidism	Euthyroidism	Hypothyroidism	p-value
CVD				
No (n, %)	0 (0.0)	128 (81.5)	29 (18.5)	0.006
Yes (n, %)	2 (6.1)	21 (63.6)	10 (30.3)	
T1DM				
No (n, %)	2 (1.1)	147 (78.2)	39 (20.7)	>0.99
Yes (n, %)	0 (0.0)	2 (100.0)	0 (0.0)	
T2DM				
No (n, %)	0 (0.0)	52 (81.3)	12 (18.8)	0.677
Yes (n, %)	2 (1.6)	97 (77.0)	27 (21.4)	
Hypertension				
No (n, %)	0 (0.0)	21 (72.4)	8 (27.6)	0.516
Yes (n, %)	2 (1.2)	128 (79.5)	31 (19.3)	
Dyslipidemia				
No (n, %)	2 (2.2)	71 (78.0)	18 (19.8)	0.493
Yes (n, %)	0 (0.0)	78 (78.8)	21 (21.2)	

## Discussion

CKD is associated with various alterations in thyroid function. A comparison of TSH, FT3, and FT4 levels across different CKD stages revealed that the incidence of hypothyroidism increased with the severity of renal dysfunction. The highest occurrence of hypothyroidism was observed in CKD stage 5, while the lowest was found in stage 1. Interestingly, patients in CKD stage 3 had a higher incidence of hypothyroidism compared to those in stage 4. This may be influenced by factors such as the type of dialysis or concurrent chronic conditions affecting thyroid function.

The impact of kidney failure on thyroid function is likely due to the kidneys’ role in iodine clearance through glomerular filtration. As kidney function declines in CKD, the GFR decreases, leading to impaired iodine clearance. This results in an elevation in plasma iodide concentrations, which increases iodide uptake by the thyroid gland. The elevated iodide levels can interfere with thyroid hormone production by inhibiting the pituitary-thyroid axis, a phenomenon known as the Wolff-Chaikoff effect. These mechanisms may contribute to the increased prevalence of hypothyroidism in CKD patients [12].

Numerous studies have documented thyroid dysfunction in CKD patients [13-15]. Consistent with our findings, a higher incidence of hypothyroidism in CKD patients has been reported in studies from Saudi Arabia [8], Japan [5,16], and India [17]. In our study, 20.5% of CKD patients had hypothyroidism, which is similar to a study conducted at SMS Medical College in Jaipur, where 26.66% of undialyzed CKD patients exhibited abnormal TSH levels, indicative of hypothyroidism or thyroid dysfunction-related to CKD [7] (Table 1). Hypothyroidism, possibly initiated by thyroid dysfunction, leads to decreased cardiac output, reduced GFR, and altered tubular function. Additionally, CKD patients are at increased risk of hypothyroidism, likely due to hemodialysis, impaired iodine clearance, or inflammatory markers like CRP and IL-6, which cause endothelial damage and affect thyroid function. Decreased FT3 production is more pronounced in advanced CKD stages, with 30.8% of stage 5 CKD patients exhibiting hypothyroidism, followed by 23.1% in stage 4. A similar study conducted in Wardha showed a significant decrease in FT3 levels as CKD progressed (p = 0.007), with the lowest FT3 levels observed in stage 5 CKD, followed by stage 4 [15]. Hemodialysis, associated with systemic acidosis, may increase inflammatory markers and endothelial dysfunction, contributing to this decline in thyroid function [13].

Furthermore, an investigation into the relationship between thyroid status and renal function parameters revealed significant associations between thyroid function and serum albumin and BUN, with p-values of 0.003 and <0.001, respectively. Elevated BUN levels were observed in patients with hyperthyroidism, while significantly lower BUN levels were found in hypothyroid patients. Our findings are consistent with a cross-sectional study of 50 CKD patients, which showed that the mean blood urea level was significantly higher than in controls (p < 0.0001). Low serum albumin levels in CKD patients suggest malnutrition [12].

Additionally, we found a significant correlation between hypothyroidism and cardiovascular comorbidities (p < 0.006). Patients with hypothyroidism had a higher prevalence of CVD (30.3%, n = 10) compared to those with euthyroidism (6.1%, n = 2). This underscores the importance of thyroid screening in CKD patients with CVD, as thyroid dysfunction may contribute to cardiovascular complications. However, no significant associations were found between TSH levels and other comorbidities, such as dyslipidemia, diabetes mellitus, or hypertension. Further research is needed to investigate the effects of sex, age, and BMI on thyroid function in CKD patients.

Two studies in Southern India (2022) and Germany (2021) demonstrated a significant linear relationship between GFR and T3 and T4 levels [18,19]. Our findings support this relationship, showing a decline in GFR alongside reductions in T3 and T4 levels. A similar cross-sectional study revealed that the prevalence of hypothyroidism in CKD patients increased as estimated GFR decreased [11].

The strengths of our study include the availability of TFT measurements (TSH, FT4, and FT3) for incident CKD events and the investigation of the correlation between CKD severity and thyroid impairment in the Saudi population. A limitation of this study is that it was based on data from a specific patient population at a single medical center, and there was a lack of information regarding patient type and dialysis duration. Additionally, the relatively small sample size limits the depth and scope of the findings, emphasizing the need for further studies with larger patient cohorts.

## Conclusions

CKD is commonly encountered in clinical practice, and individuals with CKD have an increased susceptibility to developing various medical conditions, particularly thyroid disorders. Given the high prevalence of both CKD and thyroid dysfunction, it is crucial to examine the physiological relationship between thyroid dysfunction and kidney disease. In CKD, significant alterations in thyroid function are typically seen, particularly in fluctuations of FT3 and TSH levels. Notably, the risk of thyroid dysfunction rises as renal disease severity progresses. A comprehensive understanding of these interconnections is essential for effective patient management and may inform personalized treatment strategies for individuals with both thyroid and kidney disorders. Regular monitoring and timely interventions in patients with concurrent thyroid and kidney conditions can greatly improve their overall care and prognosis.
